# Diabetes fuels periodontal lesions via GLUT1-driven macrophage inflammaging

**DOI:** 10.1038/s41368-021-00116-6

**Published:** 2021-03-24

**Authors:** Qian Wang, Lulingxiao Nie, Pengfei Zhao, Xinyi Zhou, Yi Ding, Qianming Chen, Qi Wang

**Affiliations:** 1 State Key Laboratory of Oral Diseases & National Clinical Research Center for Oral Diseases & West China Hospital of Stomatology, Sichuan University, Chengdu, China; 2 Department of Prosthodontics, West China Hospital of Stomatology, Sichuan University, Chengdu, China; 3 Department of Periodontology, West China Hospital of Stomatology, Sichuan University, Chengdu, China

**Keywords:** Immunochemistry, Cell biology

## Abstract

Hyperglycemia induces chronic low-grade inflammation (inflammaging), which is a newly identified contributor to diabetes-related tissue lesions, including the inflammatory bone loss in periodontitis. It is also a secondary senescent pattern mediated by an increased burden of senescent cells and senescence-associated secretory phenotype (SASP). Macrophage is a key SASP-spreading cell and may contribute to the maintenance of SASP response in the periodontal microenvironment. Using a transgenic diabetic model (BLKS/J-*Lepr*^*db*^/*lepr*^*db*^ mice) we identified striking senescence of the periodontium in young (18-wk)-diabetic mice accompanied by amassed p16^+^-macrophages and enhanced early SASP response. Exposed to high glucose in vitro, bone marrow-derived macrophage (BMDM) revealed a strong GLUT1 mRNA response driving the elevated-glucose uptake. GLUT1 is a representative and facilitative glucose transporter in macrophages with potential roles in hyperglycemia-induced inflammation. In this study, both GLUT1 and the downstream GTPase Rheb expression upregulated in the gingiva of diabetic mice with impaired condition. Furthermore, SASP release and p16/p21 signaling were proven to be triggered by mTOR phosphorylation in BMDM and antagonized by restricting glucose uptake in *GLUT1*^−^^*/−*^ BMDM. Taken together, our findings suggest that elevated-GLUT1 sensor responded to high glucose is important for macrophage senescence and SASP response, generated as a result of hyperglycemia, and it is a potential molecular mechanism for the exacerbation of periodontitis in diabetes.

## Introduction

Diabetes mellitus is a recognized age-related disease,^1^ and commonly is associated with chronic inflammatory bone loss in periodontitis whose pathogenesis is also concerned with age.^2^ In diabetes, enhanced inflammatory responses and the consequent tissue lesions are believed to increase the risk and severity of diabetic periodontitis.^2,3^ However, the extent to which diabetes affects periodontal diseases remains to be fully elucidated. Inflammaging, the chronic low-grade inflammation that accompanies aging, is recently implicated in the pathogenesis of diabetes-related disorders.^4,5^ During aging, cell senescence proceeds through an early senescent-response phase followed by the senescence-associated secretory phenotype (SASP) response.^5,6^ It is recognized that a heavier burden of senescent cells (SCs) together with SASP in diabetes leads to tissue disruption.^7,8^ The activity of Interleukin (IL)−1β represents the early SASP response,^9^ one of the specific determinants of aging.^10,11^

Macrophage is the master SASP-carrying cell^12^ as well as the essential immune cell in the periodontal tissue defense.^13^ In diabetes, hyperglycemia activates pro-inflammatory phenotypes in macrophages, which has an integral role in the promotion of hyperglycemia-induced inflammation.^12^ Similar to cells in other organs, macrophage also becomes senescent or exhausted during aging, which in turn contributes to a systemic low-grade chronic inflammation and tissue lesions. High-glucose intake and transport are tightly linked to inflammatory responses as well as cellular senescence through metabolic pathways.^12,14^ Increasing SCs burden in diabetic mice has been reported to be affiliated with deteriorative glucose tolerance, more accumulation of macrophage, and elevated production of pro‐inflammatory phenotypes.^15^

The glucose transporter 1 (GLUT1) is an important representative of facilitative glucose transporter in macrophages.^16,17^ Growing evidence points to GLUT1-dependent glycolmetabolic activation in the pathological injury of the aged tissue.^18^ The overexpression of GLUT1 induced by hyperglycemia promotes immuno-function macrophage and expands its infiltration.^19^ Inhibition of GLUT1 provides a potential target of therapy to diabetic complications.^20,21^ In this study, we focused on the role of inflammaging that occurred in diabetes on periodontal tissue damage, and investigated the potential mechanism of GLUT1 in hyperglycemia-induced macrophage senescence and SASP response in the periodontal tissue.

## Results

### Hyperglycemia leads to a SASP response in the periodontium of diabetic mice

**Fig. 1:**
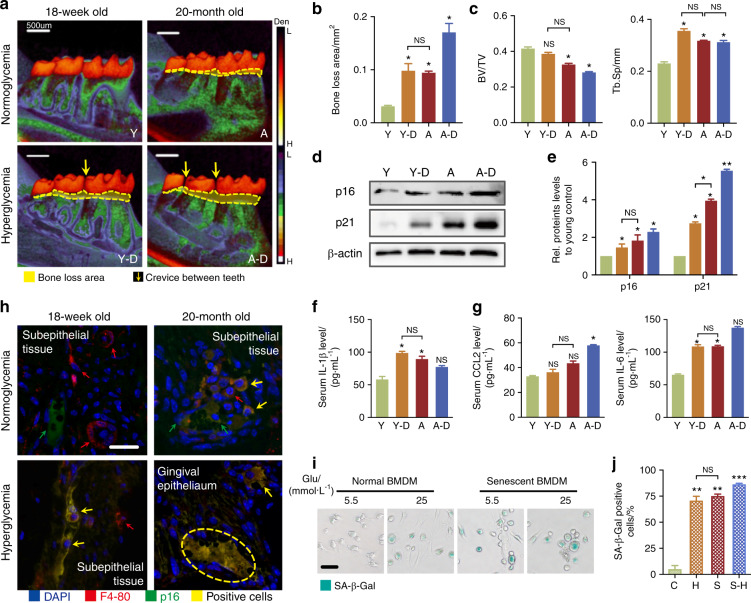
Activation of p16/p21 and SASP in periodontal lesions of diabetic and aged mice. **a** Periodontal damage in diabetic and aged mice. Micro-CT analysis showed the periodontal damage. The area of bone loss was shown by the area of density reduction below the crown. The alveolar bone resorption area is indicated by yellow line areas. Scale bar: 500 μm. **b** Quantification of alveolar bone resorption area (the area of density reduction below the crown). **c** Quantification of bone volume/total volume (BV/TV) and Tb.Sp (trabecular separation). **d** p16 and p21 were detected by western blot. **e** Protein p16/p21 levels relative to β‐actin protein levels were assessed by densitometric analysis and expressed as a percentage of the levels of wild‐type mice. **f**, **g** Serum early/late SASP response in diabetic and aged mice. The SASP responses (Supplementary Table 2) were detected by ELISA, including early SASP (IL-1β) and late SASP response (CCL2 and IL-6). **h** The location of p16^+^ macrophage in periodontal lesions. Immunohistochemistry staining was applied to co-localize p16 and F4-80 (macrophage mark) protein expression in periodontal tissues. Note the bright p16 flake in cells that co-localized with a prominent flake of F4-80. The yellow arrow indicated the double-positive cells. The red arrow indicated the F4-80^+^-positive cells and the green arrow indicated the p16^+^-positive cells. Scale bar = 20 μm. **i**, **j** Features and quantification of high-glucose-induced and drug-induced (DIS) senescent BMDMs. SA-β-Gal staining was used to display the senescent cells. The green represented activation of senescence. Scale bar: 50 μm. Y, young normoglycemic mice (18-wk-old); D, young-diabetic mice; A, aged normoglycemic mice (20-mo-old); A–D, aged-diabetic mice. C, BMDM cultured in low-glucose (5.5 mmol·L^−1^) condition; H, BMDM cultured in high-glucose (25 mmol·L^−1^) condition; S, DIS-BMDM cultured in low-glucose condition; SH, DIS-BMDM cultured in high-glucose condition. *P < 0.05. **P < 0.01. ***P < 0.001. NS, no significance. Repeated three times. Data are presented as the mean ± SD (*n* = 10) and compared to the normal

To understand the reason that hyperglycemia aggravates periodontal damage, we started with a contrastive study in young (18-week-old) mice versus aged (20-month-old) mice treated with or without streptozotocin (STZ). Young-diabetic mice showed similar obvious inflammatory bone loss (Fig. 1a–c) and induction of p16/p21 in periodontal tissues to aged mice (Fig. 1d, e). The periodontal condition in aged-diabetic mice was worse than the symptoms in diabetic-only or aged-only mice (Fig. 1a–c). These manifestations indicated that diabetes indeed exacerbates periodontal inflammation, as reported. IL-β, a hallmark of early SASP response, showed a 2-fold, 1.8-fold, and 1.4-fold increase in diabetic, aged, and aged-diabetic mice, respectively (Fig. 1f). However, the magnitude of the later SASP response (featured as induction of c-c motif chemokine ligand 2, CCL2; MMP3)^9^ was not significantly amplified in diabetic mice, except for IL-6 (Fig. 1g and Supplementary Fig. S1a). Similar to the aged group, the p16^+^-macrophage was found in periodontal tissues of diabetic mice, and even more in the aged-diabetic group (Fig. 1h and Supplementary Fig. S1b). To further characterize the senescent/inflammatory response in high-glucose conditions, a drug-induced senescent (DIS) cell model was applied (Fig. 1i, j). Interestingly, elevated senescence-associated β-galactosidase (SA-β-Gal) activity, induced the protein p16/p21 expression and the early SASP response was observed in high glucose, like the appearance in DIS and high-glucose DIS-BMDM (82%, 87%, and 97.2%) (Supplementary Fig. S1c–f). These results prompted that diabetes intensified the early SASP response and pro-inflammatory/senescent phenotypes of macrophages and may be the cause of periodontal damage resulted from hyperglycemia.

### Young-diabetic mice display macrophage senescence in periodontal lesions

**Fig. 2:**
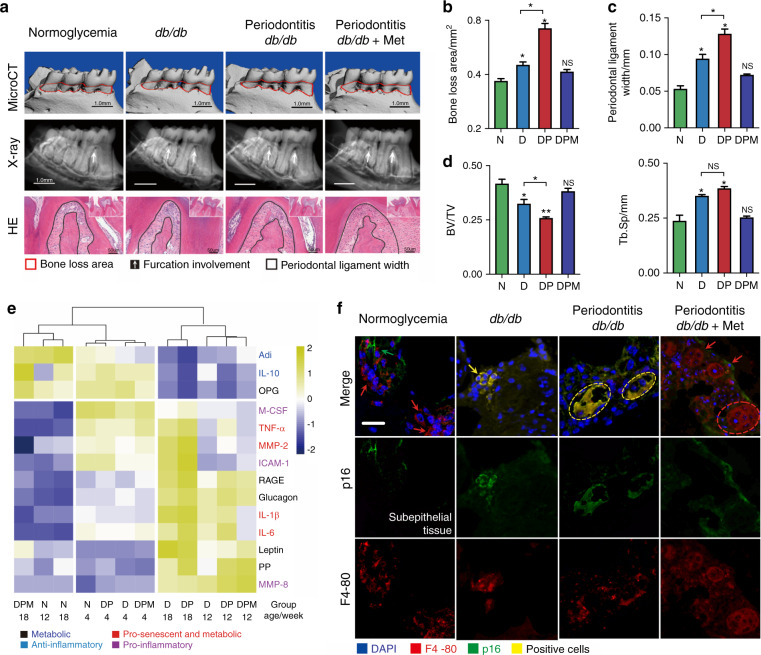
Activation of p16/p21 and SASP in periodontal lesions of young *db*/*db* mice. **a** Periodontal damage in 18-wk-old *db/db* mice. Top row: The 3D-reconstructed mandible in different groups. Vertical bone resorption is indicated by a red dotted area. Middle row: The Furcation involvement indicated by the white arrow was shown by X-ray observation. Bottom row: The width of the periodontal ligament is indicated by black line areas. **b** Quantification of alveolar bone resorption area and CEJ-ABC length: distance from the cement-enamel junction (CEJ) to the alveolar bone crest (ABC). Measurements are expressed in mm^2^. **c** The vertical distance from the top of the alveolar ridge to the bottom of root furcation was used to quantify the periodontal ligament width. **d** Quantification of BV/TV and Tb.Sp. **e** The serum SASP response in young *db*/*db* mice. The heat map cluster represented the serum SASP profile for different groups at 12- and 18-wk old (Supplementary Table 3), including IL-1β, IL-6, IL-10, TNF-α, MMP-2, MMP-8, ICAM-1, M-CSF, RAGE, PP, glucagon, leptin, Adi, and OPG. **f** Location of p16^+^-macrophage (F4-80^+^) in periodontal lesions of young *db*/*db* mice. The yellow arrows presented the double-positive cells, the red for F4-80^+^ cells, and the green for p16^+^ cells. Scale bar: 20 μm. N, normoglycemic group; D, *db*/*db* group; DP, periodontitis-*db*/*db* group; DPM, metformin-treated periodontitis-*db*/*db* group. *P < 0.05. **P < 0.01. NS, no significance. Repeated three times. Data are presented as the mean ± SD (*n* = 10) and compared to the normal

To gain insight into the role of hyperglycemia in periodontal inflammation, we focused on the young-diabetic mice and the established periodontitis-diabetic mice. As we previously reported,^22^ diabetic (D) and periodontitis-diabetic (DP) groups showed uncontrolled body over-weight gain and increased glycemia markedly (Supplementary Fig. S2a, b). A characteristic feature of periodontitis is the loss of bone around the teeth. Like diabetic humans, *db*/*db* mice had naturally occurring spontaneous periodontal bone resorption, leading to furcation involvement and wide periodontal ligament (Fig. 2a–d). Periodontal bone loss typically reflects the degree of inflammation. Quantitative analysis of the micro-CT results demonstrated that the bone loss of *db*/*db* mice with *P.g*. infection was nearly double compared to the normoglycemia (N) (Fig. 2b). Histochemistry analysis was carried out increased p16 and p21 proteins in the periodontal tissues of *db*/*db* and periodontitis-*db*/*db* mice (Supplementary Fig. S2c, d). The comprehensive SASPs panels gave similar results, that majority of cytokines inducing bone resorption upregulated in the D and DP mice (Fig. 2e). The differences became more obvious in 12-week-old mice, including macrophage-specific phenotypes, pro-senescent phenotypes (tumor necrosis factor (TNF)-α), and a distinct metabolic phenotype (receptor for the advanced glycation end product (RAGE), Glucagon and IL-10).^11^ The induction of pro-senescent and metabolic phenotypes was also reported to be involved in periodontal inflammation.^10^ The SASP response peaked at the 18th week in *db*/*db* mice with *P.g*. infection (Fig. 2e), indicating that bacteria fostered SASP release under the diabetic condition. Diabetes caused a 5-fold increase in the number of macrophages expressing p16 in the periodontium. In the periodontal lesions of the periodontitis-*db*/*db*, the co-localization of p16/F4-80 has the largest increase of cells examined, which was consistent with the presence of aged mice (Fig. 2f and Supplementary Fig. S2e). Metformin exerts a protective effect by relieving the worse condition triggered in diabetes (Fig. 2a–f).

### GLUT1 promotes macrophage senescence/inflammation in diabetic-periodontal lesions

**Fig. 3:**
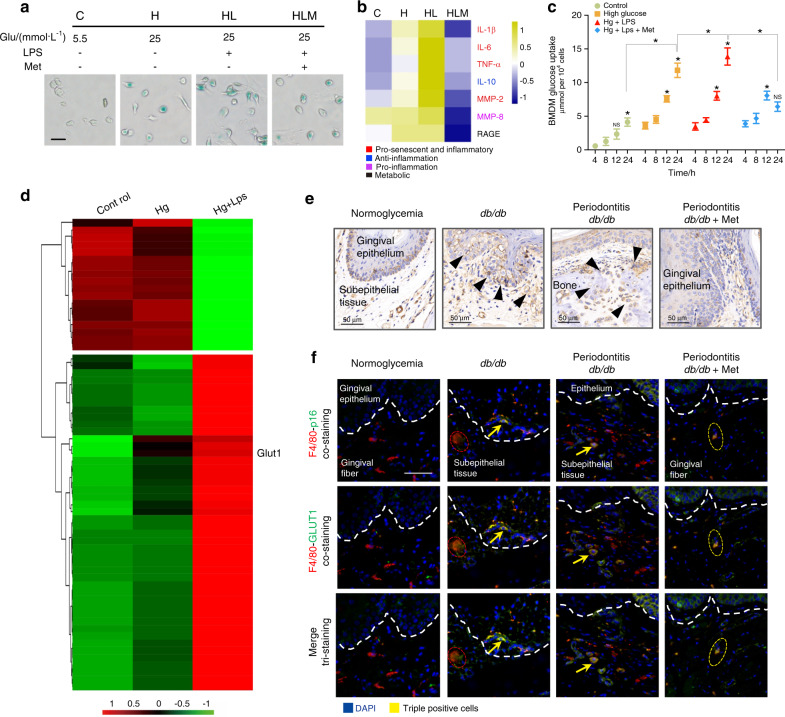
Overexpression of GLUT1 protein modulates macrophage inflammation/senescence. **a** SA-β-Gal staining was used to display the senescent cells. The green represented activation of senescence in BMDM. Scale bar: 50 μm. **b** Induction of SASP response in vitro. Heat map cluster was shown the SASP profiles in BMDM supernatant, including distinct inflammatory, pro-senescent and metabolic phenotypes. **c** The glucose uptake of macrophage in vitro. The glucose uptake of BMDM at 4-, 8-, 12-, and 24-h glucose stimulation, respectively. The concentration was expressed in μmol per 10^4^ cells. **d** Accumulation of glycometabolic/inflammatory genes. Heat map representation showing the relative abundance (expressed as *Z*-scores) for the 63 genes significantly changing expression in high-glucose BMDM (Supplementary Table 1). Column clustering was calculated as a 1-Pearson correlation. Rows have been grouped into functional subsets of genes. **e** GLUT1 expression in periodontal lesions by IHC staining. The positive cells are indicated by black arrows. Scale bars: 50 μm, ×40. **f** High levels of GLUT1 in p16^+^ macrophage of *db*/*db* mice. IF staining for co‐localizing p16/GLUT1/F4-80 in periodontitis lesions showed the triple-stained positive cells. The positively stained cells are indicated by yellow arrows. Bars: 50 μm. C, 5.5 mmol·L^−1^ -glucose BMDM; H, 25 mmol·L^−1^ glucose-BMDM; HL, 25 mmol·L^−1^ glucose-BMDM stimulated with LPS; HLM, metformin-treated HL-BMDM; N, normoglycemic; D, *db*/*db*; DP, periodontitis-*db*/*db*; DPM, metformin-treated periodontitis-*db*/*db*. *P < 0.05. **P < 0.01. Repeated three times. Data are presented as the mean ± SD (*n* = 10) and compared to the normal

Results above indicate that the transition to hyperglycemia in diabetic mice induces a change in periodontal condition, periodontal inflammation, and macrophage senescent state. These changes are indicative of a more pronounced pathogenic environment in the diabetic group. To determine whether this could be accounted for by hyperglycemia-induced macrophage senescence/inflammation alone, we further investigated the glycometabolism of macrophages in vitro. Same as DIS-macrophage, SA-β-Gal activation increased strongly in 25 mmol·L^−1^ glucose (80.9%), especially plus 12-h LPS stimulation (Fig. 3a and Supplementary Fig. S3a). Enhanced p16/p21 was confirmed in 25 mmol·L^−1^-glucose groups relative to the control (Supplementary Fig. S3b, c), in line with the presentation of macrophage in diabetic periodontium (Supplementary Fig. S2c, d). Higher SASP response was detected in 25 mmol·L^−1^-glucose BMDM supernatant than that of 5.5 mmol·L^−1^-glucose BMDM (Fig. 3b). Interestingly, the glucose sensitivity of high-glucose BMDM increased drastically compared to the normal glucose, with a higher glucose uptake after LPS stimulation (Fig. 3c). Metabolism reprogramming of macrophages could drive a pro-inflammatory/pro-senescence phenotype.^17^ Micro-array analysis revealed a significant difference between the high glucose and the control in 63 biochemicals, in which *GLUT1* mRNA accumulated significantly (Fig. 3d and Supplementary Table 1). Inflammatory response, positive regulation of cell proliferation, and metabolic pathways presented predominantly in high-glucose BMDM (Supplementary Fig. S3d, e). These results suggest that macrophage senescence, as well as inflammation triggered by high glucose possibly, relates to its glycolmetabolic reprogramming. As we observed in vitro, the IHC staining displayed that GLUT1 was elicited upregulation (57.8% vs. 36% of normoglycemic mice) in the diabetic lesions (Fig. 3e and Supplementary Fig. S3f). Co-localization for GLUT1/F4-80/p16 showed widespread triple-positive cells in the periodontal lesions of *db*/*db* mice (15.7% vs. 0.9% of normoglycemic mice) (Fig. 3f and Supplementary Fig. S3g), indicating that GLUT1 overexpression might inhibit macrophage cycle and contribute to macrophage inflammation in diabetes-related periodontal lesions.

### GLUT1/mTOR signal modulates hyperglycemia-induced senescence/inflammation in macrophage

**Fig. 4:**
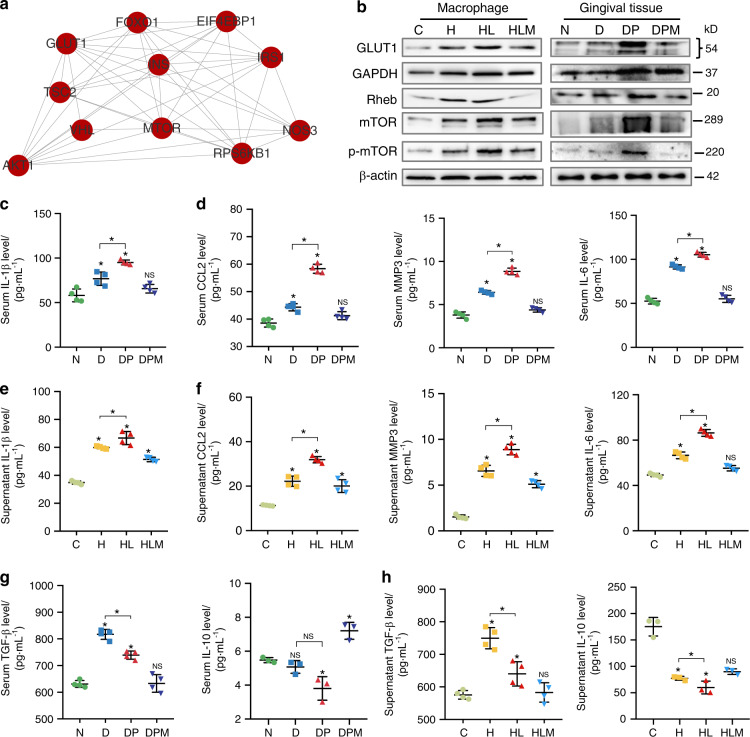
GLUT1/mTOR pathway modulates hyperglycemia-induced senescence and inflammation. **a** The protein–protein interaction network (PPI) displayed the interaction between *GLUT1* and *mtor*. **b** The expressions of GLUT–mTOR pathway proteins in vivo and in vitro. Western blot was applied to detect the expression of GLUT1, mTOR, GAPDH, Rheb, and phosphorylated-mTOR (p-mTOR) in BMDM and the gingival tissues of different groups. **c**–**h** The downstream early/late SASP response. The SASP responses in vivo (**c**, **d**, **g**) and in vitro (**e**, **f**, **h**) were detected by ELISA, including early SASP (IL-1β), late SASP response (CCL2, MMP3, and IL-6), and metabolic SASP (IL-10 and TGF-β). C, BMDM cultured in 5.5 mmol·L^−1^ glucose; H, 25 mmol·L^−1^ glucose-BMDM; HL, 25 mmol·L^−1^ glucose-BMDM stimulated with 12-h LPS; HLM, HL-BMDM administrated with metformin for 12 h; N, normoglycemic mice; D, *db*/*db* mice; DP, periodontitis-*db*/*db* mice; DPM, metformin-treated periodontitis-*db*/*db* mice. *P < 0.05. **P < 0.01. ***P < 0.001. Repeated three times. Data are presented as the mean ± SD (*n* = 10) and compared to the normal

To investigate the underlying mechanism, we next determined which downstream regulatory proteins of GLUT1 are involved in senescent/inflammatory induction by hyperglycemia. PPI analysis revealed that GLUT1 activity might have a strong bearing on mammalian target of rapamycin (mTOR) signaling (Fig. 4a and Supplementary Fig. S4a). GLUT1 effected on mTOR activity has been reportedly linked to the reduced interaction between GTPase Rheb and glyceraldehyde-3-phosphate dehydrogenase (an enzyme providing energon H^+^ in glycometabolism, GAPDH).^23^ mTOR signaling has a positive regulation in signal transduction, overlapping with senescence response in diabetic pathology.^24^ Notably, GAPDH, Rheb, and p-mTOR expressions were upregulated in the diabetic gingiva, in line with the results in vitro (Fig. 4b and Supplementary Fig. S4b). Given that nuclear factor kappa-light-chain-enhancer of activated B cells (NF-κB) was upregulated (Supplementary Fig. S4c, d), the main transcription platform controlling SASP secretion,^25^ p-NF-kB/IL-1β expression was evaluated. Both downstream proteins enhanced progressively in the diabetic gingiva (Supplementary Fig. S4c). GLUT1 signaling augment was coordinated with early but not late SASP markers (Fig. 4c–f), including metabolic phenotypes (IL-10 and transforming growth factor (TGF)-β) (Fig. 4g, h). This further demonstrates that GLUT1–mTOR signaling contributes to the pre-establishment of a mature SASP response in macrophages.

### Hyperglycemia-induced inflammaging is alleviated in *GLUT1*^−/−^ macrophage

**Fig. 5:**
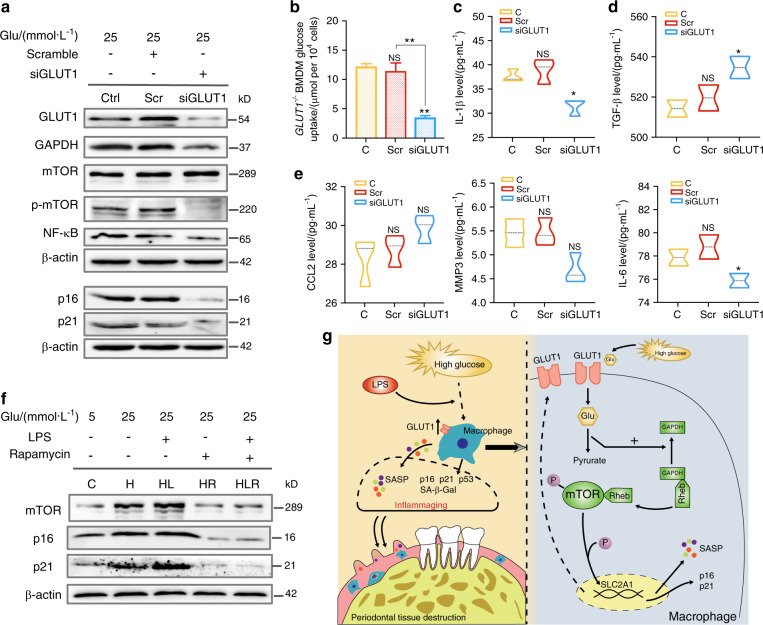
Induction of p16/p21 and SASP response by high glucose is relieved in GLUT1^−/−^ macrophage. **a** GLUT1–mTOR pathway proteins expression in GLUT1-knockout BMDM. Small interfering RNA (siRNA) targeting mouse GLUT1 was applied to build a *GLUT1*^−/−^ BMDM model. The non-targeted siRNA as a negative control (Scramble). **b** Glucose uptake of *GLUT1*^−^^*/−*^ BMDM. **c**–**e** The downstream SASP response in *GLUT1*^−^^*/−*^ BMDM. The SASP response in *GLUT1*^−^^*/−*^ BMDM was detected by ELISA, including early SASP (IL-1β), late SASP (CCL2, MMP3, and IL-6), and metabolic SASP (TGF-β). The concentration was expressed in pg·mL^−1^. **f** The mTOR downstream protein in BMDM treated with mTOR inhibitor, rapamycin, was detected by western blot. **g** Proposed model of mechanisms showed that macrophage inflammaging promotes periodontal damage by increasing the GLUT1 sensor response to high glucose and activating the mTOR pathway. C, BMDM cultured in low-glucose medium (5.5 mmol·L^−1^); H/HL, BMDM cultured in high-glucose medium (25 mmol·L^−1^) without or with LPS; H-R/HL-R, BMDM cultured in 12-h H/HL condition and then treated with 50 μg·L^−1^ rapamycin. *P < 0.05. **P < 0.01. ***P < 0.001. NS, no significance. Repeated three times. Data are presented as the mean ± SD (*n* = 5)

To further identify the role of GLUT1–mTOR in hyperglycemia-induced inflammaging, siRNA targeting GLUT1 was introduced into BMDM. Glucose uptake, as well as GAPDH expression, were dampened in *GLUT1*^−/−^ macrophage (Fig. 5a, b and Supplementary Fig. S5a, b). However, GLUT1 inhibition had a marked effect on p-mTOR rather than mTOR expression (Fig. 5a and Supplementary Fig. S5c), revealing that GLUT1 activity might involve in mTOR phosphorylation. Effective ablation of NF-kB and p16/p21 signaling was achieved in *GLUT1*^−/−^ macrophage (Fig. 5a and Supplementary Fig. S5d–f). In parallel, the downstream SASP response markedly declined in *GLUT1*^−/−^ macrophage (Fig. 5c–e), especially early and metabolic SASP levels ameliorated but not including representative late SASPs (Fig. 5c, d). As predicted by metformin in vivo, inhibiting mTOR signaling with rapamycin weakened p16/p21 induction in BMDM (Fig. 5f and Supplementary Fig. S5g–i).

## Discussion

Diabetic complications are related to inflammation, where hyperglycemia is one of the major determinants.^3,4^ In this study, we highlighted a potential association between periodontal lesions and hyperglycemia-induced inflammation and senescence (Fig. 5g). Diffused macrophage-cycle inhibition and early SASP responses developed in periodontal tissues of diabetic mice and aged mice. Hyperglycemia triggered macrophage inflammation/senescence through the altered glycometabolism mediated by GLUT1, which might accelerate periodontal damage.

Macrophage senescence is an essential feature of immune dysfunction in response to a variety of stresses triggered by glucolipotoxicity.^26^ Immuno-functional macrophages might collapse and worsen to senescence under high-glucose stimulation. Diabetic mice acquired a senescent phenotype like the aged mice, implying that hyperglycemia might accelerate aging by inflammation. p21 and p16^INK4a^, cyclin‐dependent kinase inhibitors, during physiological aging and several age-related diseases directly implicate this well‐established effector of senescence in the aging process.^27,28^ There have been reports^29^ underscored that the elimination of p16^Ink4a^‐expressing cells from mice increased lifespan and ameliorated a range of age‐dependent/disease‐related abnormalities, suggesting that the accumulation of p16^Ink4a^‐expressing cells during aging shortens lifespan.^27^ High glucose displayed increased p16/p21, activated SA-β-Gal, and elicited SASP response, consistent with in vivo data for diabetic mice. It suggests a mechanism whereby high glucose stimulates inflammation in BMDM, which may account for the increased macrophage senescence and periodontal inflammation observed in diabetic mice. Oral pathobiont-reactive macrophages that arise during oral inflammation contribute to the activation and infiltration of other inflammatory cells inducing tissue destruction, such as neutrophils.^30^ Notably, senescence spreads at the systemic level, further fueling chronic inflammation and aggravating periodontal destruction.^31,32^

Cell senescence is now known to be involved in the pathogenesis of diabetes by subsequent SASP response. SASP response reinforces the senescent phenotypes by acting in an autocrine and paracrine manner.^7^ The majority of SASP is released into tissue and circulation by macrophages.^33^ These factors triggered the upregulation of primary periodontal tissue destruction mediators in diabetes, even altered oral bacterium.^31^ The latest literature^9^ pointed that early SASP response, characterized by the upregulation of IL-1β, is the main feature of cellular senescence. IL-1β also is a hallmark of the periodontal inflammatory state.^34^ IL-1β, pro-senescent SASP, and metabolic phenotypes were elevated in diabetic mice sera, which together support the cellular senescence in the diabetic periodontium. Along with the senescence deterioration, late SASP response appears to exacerbate periodontal lesions. This low-grade inflammation triggers more SASP activation in macrophage, mostly secondary to metabolic factors through a process of glucolipotoxicity.^35^

Periodontitis is a chronic inflammatory disease in which bacterial infection and host inflammatory responses are two main pathogenesis.^3,36^ Diabetes was prone to discrepant periodontal damage, which may attribute to aberrant host inflammatory responses caused by hyperglycemia.^3,37^ Our previous published paper^38^ also revealed obvious periodontal damage in diabetic mice similar to *p.g.-*infected non-diabetic periodontitis mice. The literatures^3,36^ have reported the potential impact of oral-microbiome change in diabetic conditions. In our study, *p.g.-*infected diabetic mice performed an extent SASP response, also suggesting that the oral-microbiome remold may cooperate with hyperglycemia to magnify the periodontal inflammatory status. It seems to be a vicious-feedback loop in which hyperglycemia has increased levels of periodontal SASP response, and the increased gingival macrophage inflammation and/or senescence, in turn, induces alterations in the bacteria to make it more inflammatory.^3^ Additionally, oral-microbiome change fuels systemic inflammation (inflammaging)^31^ through intermittent interaction between bacteria and macrophage, which in turn enhances insulin resistance to promote hyperglycemia.

The mechanism of hyperglycemia accelerating macrophage inflammation has not been clarified. Glucose is the primary fuel metabolized in senescent/pro-inflammatory macrophages.^16^ Elevated GLUT1-driven glycometabolism spurs pro-inflammatory mediator expressions in macrophages,^17^ which is a possible source of SASP in hyperglycemia-induced inflammation. These SASP could rectify the primary- and meta-inflammation in hyperglycemia-induced inflammation, linking several researches with observations in other diabetic organs.^39^ Macrophage in periodontal inflammation has high transcriptional activity as well as metabolic demand; hyper-glycometabolism was needed to rectify this phenomenon, however, this could lead to macrophage exhaustion and a return to senescence. A plethora of pathways have been implicated in SASP and cellular senescence regulation, including NF-κB, mTOR, and NLR family.^5^ mTOR is one of the master modulators of SASP and the aging rate.^40^ Levels of GLUT1 expression and mTOR activation, as evidenced by S6 kinase (S6K) and 4E-BP-1 phosphorylation, changed in tandem in cell lines exposed to elevated levels of extracellular glucose.^23^ Rheb, a GTP-binding protein modulated by tuberous sclerosis complex (TSC), activates and regulates mTOR activity. When bound to GTP, Rheb activates mTOR activity.^23,41^ *GLUT1*^−/−^ macrophage abrogated the active effect of Rheb on mTOR, which verified that high glucose-induced macrophage inflammation is dependent on GLUT1 and mTOR effected on inflammaging is triggered by glycometabolism. Gene profiling in SCs treated with metformin pointed that metformin reduced the pro-inflammatory cytokines expression, mostly regulated by mTOR.^40,42^ Specifically, SASP such as TNF-α, IL-1β, and IL-6 triggered the upregulation of primary periodontal tissue destruction mediators in diabetes,^34^ which was markedly decreased by metformin.

In summary, there is a complex interaction between diabetes, inflammation, and periodontal disease including the oral microbiome. We sought to dissect each component separately by detecting a senescent phenotype in the young-diabetic periodontium and by examining senescence and SASP response through high-glucose stimulation. These results suggest that hyperglycemia-induced cellular senescence and inflammation interact with each other and each contributes to the susceptibility and severity of periodontal disease with diabetes. There are a few limitations of our study that young *db*/*db* mice on a BLK background while the aged-diabetic mice are BL6 background. C57BL/6J mice with STZ-induced diabetes kept for 20 months were used as aged-diabetic model^43^ because that BLK^*db*/*db*^ mice obtaining uncontrollable hyperglycemia survived only for 10 months.^44^ Within the limitation of the study, these results implied that GLUT1-mediated macrophage inflammation and SASP response might have vital roles in diabetes-related periodontal lesions. Although this study demonstrated based on the previous findings of the Rheb linking GLUT1 and mTOR in macrophage glycometabolic reprogramming, it is worthy of more in-depth study.

## Methods

### Animals

The 4-week and 17-month-old male C57BL/6J wild-type mice were injected with STZ (50 mg·kg^−1^/3d) to establish young/aged-diabetic models.^43^ After 6 h, and until 24 h after STZ injection, STZ-injected mice in the group 2/4 were provided with 10% glucose solution to prevent diabetogenic-induced hypoglycemia.^45^ After five to eight STZ injections mice with non-fasting blood glucose levels = 250 mg·dL^−1^ were chosen for the study. The mice were divided into four groups for the experiment (10 mice per group) and kept for 3 months:

(1, 2) young mice (4-week-old) with or without STZ-induced diabetes (Y and YD);

(3, 4) aged mice (17-month-old) with or without STZ-induced diabetes (A and AD).

The 4-week-old male *db*/*db* (C57BL/KsJ-Lepr^*db*^/lepr^*db*^) mice were acquired from the Model Animal Research Center of Nanjing University for mating and breeding to establish another young-diabetic model. They developed spontaneous T2DM with sustained hyperglycemia^46^ but experienced no incident of severe hyperglycemia.^47^ The experimental groups as follow (10 mice per group):

(5, 6) normoglycemic groups (C57BL/KJ) and *db*/*db* mice (N and D);

(7, 8) bacteria-infected *db*/*db* mice with or without metformin treatment (DP and DPM).

All mice were handled in the State Key Laboratory of Oral Diseases at Sichuan University under standard housing conditions and were in accordance with the protocol approved by the institutional committee for animal use and care at Sichuan University (No. WCCSIRB-D-2015-075).

### Bacterial infection and administration of metformin

The mouse model of periodontitis was established according to the previous protocol.^47^ *Porphyromonas gingivalis* (*p.g*., ATCC 33277), which is the most common pathogen suspected of inducing periodontitis, was provided by the State Key Laboratory of Oral Diseases of Sichuan University. As we published previously,^22^ *P.g*. was grown anaerobically in sheep-blood agar with hemin/vitamin K1 for a week. After three passages of cultivation, the pure culture was inoculated in a liquid brain heart infusion broth medium and cultured for 48 h at 37 °C anaerobically. The DP and DPM mice at 5-wk age were infected with *p.g*. orally (10^9^ live bacterial cells and 2% (w/v) carboxymethylcellulose in 100 μL of phosphate-buffered vehicle, three times at 2 d intervals) and then the control was administered with vehicle. From a 7-week old, mice in the DPM group received metformin (oral intubation,^22^ 200 mg·kg^−1^ d^−1^, Sigma-Aldrich, USA). Mice in the other groups received a mean volume of sterile water. The administration was continued to 1 day before killing.

### Micro-CT analysis

After disassociating the attached soft tissue, the mandibular jaws of all groups were scanned by a µCT50 (SCANCO Medical, Bruettisellen, Switzerland) at 10 μm intervals. The alveolar bone was defined as the region of interest (ROI) with a spatial resolution of 7 μm.^48^ According to the ROI, bone volume/total volume (BV/TV), trabecular number (Tb.N), and trabecular separation (Tb.Sp) were calculated for quantification. As described previously,^49^ we captured all images of these mandibular samples by 3D reconstruction to analyze the periodontal bone loss. Measurements are expressed in mm^2^. According to the different densities of the 3D reconstruction mandibular (Fig. 1a), the dual-channel analysis method was applied, and the channel was selected for the crown (PET 20) and the other parts (Heat). The area of bone loss was shown by the area of density reduction below the crown. In addition, the X-ray observation of bone resorption in the furcation area also was shown the severity of alveolar bone loss in different groups.

### Biochemical analysis

Mouse tail vein blood was collected following a 10-h fast. A glucometer (OneTouch Glucometer; LifeScan, Milpitas, CA) was subsequently used to determine the blood glucose level every 2 weeks. Meanwhile, the tail vein blood of each mouse was collected by the capillary pipette from 4 to 18 weeks old, then serum was directly isolated (centrifuging at 3 000 × *g* for 10 min) and stored at −80 °C. The SASP levels were determined using Luminex multiple-factor assay kits (Luminex 200, R&D Systems, Minneapolis, MN, USA) according to the manufacturer’s specifications. SASP-specific changes between cohorts were visualized by row-specific *Z*-scores in a heat map. A glucose assay kit (ZC-S0418; ZCI BIO, China) was used to detect the glucose uptake of BMDMs (μmol per 10^4^ cells). ELISA analysis was used to detect the levels of early/late SASP (Supplementary Table 4).

### Western blotting

To harvest total protein from mouse gingival tissues, the soft tissues around the mandibular molars were carefully scraped out. Total protein was extracted according to the manufacturer’s specifications. The proteins were subjected to 5% (w/v) sodium dodecyl sulfate–polyacrylamide gel electrophoresis gel and transferred to nitrocellulose membranes by electro-blotting. The membranes were incubated overnight at 4 °C with primary antibodies (Supplementary Table 4) then with secondary antibodies for 1 h at room temperature. The signals were detected by electrochemiluminescence using a Bio-Rad system (Bio-Rad Laboratories, Shanghai, China). For quantitation, phosphorylated proteins were normalized to the levels of the total (phosphorylated + non-phosphorylated) protein and other proteins were accomplished by normalizing to β-actin.

### Senescence-associated β-galactosidase (SA-β-Gal)

SA-β-Gal staining was performed using SA-β-Gal staining kit (C0602; Beyotime Bio., China) according to the manufacturer’s instructions. After being rinsed with PBS, cells were observed for the blue staining with a microscope at ×20 magnification.

### Cell models

Bone marrow-derived macrophage (BMDM) was obtained from 6-week-old male *db*/*db* mice bone marrow and cultured in DMEM medium (Gibco, USA) with 20% (v/v) FBS, 1% penicillin-streptomycin, 50 ng·mL^−1^ M-CSF (PeproTech, USA) for 1 week to mature.^22^ As described previously^50^ a drug-induced senescent (DIS) cell model established, 500 μmol L^−1^ hydrogen peroxide (H_2_O_2_) was applied to stimulate BMDM in low-glucose (5.5 mmol·L^−1^ d-glucose) or high-glucose condition (25 mmol·L^−1^) for 24 h. The senescent cells were verified to be well-established by senescence-related tests (p16/p21 cell-cycle inhibitor and SA-β gal activity). LPS (Sigma-Aldrich Trading Co. Ltd., St. Louis, MO, USA) from *p.g*. was applied to simulate infection‐induced periodontal inflammatory conditions. BMDM was co-cultured with LPS and TNF-α (10 ng·mL^−1^, a pro-inflammatory agent mediating senescence-driven periodontal tissue dysfunction in diabetes) to mimic the periodontal inflammation under diabetes.^51^ Cells were cultured at 37 °C in a humidified atmosphere containing 5% CO_2_.

### Histologic and immunohistochemical analyses

To investigate general morphology, the maxillary samples were coronally incised (4 μm) and subjected to stain with hematoxylin and eosin. Immunofluorescent staining was performed to co‐localize p16, F4/80, and GLUT1 in the periodontal tissues. The sections were incubated with p16 (Cat# sc-166760, Santa Cruz Biotechnology, USA), F4/80 (Cat# 30325, Cell Signaling Technology, USA), and GLUT1 (Cat# ab40084, Abcam, USA) primary antibodies, followed by Alexa Fluor 555-conjugated secondary antibodies (Cat# A32727, Cat# A32732, Invitrogen, USA). The sections were then stained with DAPI (Cat# 28718-90-3, Sigma-Aldrich, USA) to locate the nuclei. The number of p16‐F4/80 double-, F4/80-p16-GLUT1 triple-cells across five randomly selected regions was counted with a microscope and expressed as a percentage of the total number of cells.^52^ Immunohistochemistry analysis was used according to the manufacturer’s instructions with the following antibodies: mouse monoclonal p16 and p21 (Cat# 9176, Cat# 9145, Cell Signaling Technology, USA).

### RNA extraction

Total RNA was extracted from BMDM cultured with different processing conditions based on the study design, using Trizol reagent (Invitrogen Life Technologies). RNA was quantified by the NanoDrop ND-2000 (Thermo Scientific) and the RNA integrity was assessed using Agilent Bioanalyzer 2100 (Agilent Technologies).

### Microarray analysis

The sample labeling, microarray hybridization, and washing were performed based on the manufacturer’s standard protocols. The arrays were scanned by the Agilent Scanner G2505C (Agilent Technologies). Feature Extraction software (V. 10.7.1.1) was used to analyze array images to get raw data. Genespring (V. 14.8) was employed to finish the basic analysis with the raw data. Differentially expressed mRNA was then identified through fold change (≥1.0) as well as *P*-value (≤0.05) calculated with *t*-test. Difference integration analysis (Venn analysis) was then performed (Supplementary Table 1). The high-frequency characteristic elements between the three groups were determined by Venn analysis. Differentially expressed mRNAs were analyzed using Cluster software (V. 3.0). GO (Gene Ontology) and KEGG analysis (Database for Annotation, Visualization, and Integrated Discovery) was applied to determine the roles of these co-expressed genes. GO terms with *P*-value < 0.05 were selected and integrated using Venn analysis. The top 63 enriched often GO terms among the three groups ranked by fold enrichment and enrichment score were presented. KEGG analysis was performed to determine the involvement of co-expressed genes in different biological pathways. The protein–protein interaction network (PPI) was displayed the interaction between *GLUT1* and *mtor*.

### GLUT1 knockdown

Small interfering RNA (siRNA) targeting mouse *GLUT1* designed according to reference^20^ (5′-GGAATTCAATGCTGATGATGA-3′, 5′-TCATCATCAGCATTGAATTCC-3′, GenePharma Co.) was used to block GLUT1 in BMDM. Non-targeted siRNA as a negative control (5′-TTCTCCGAACGTGTCACGT-3′, 5′-ACGTGACACGTTCGGAGAA-3′) was used in wells not transfected with *GLUT1* siRNA. Normal saline-treated with diethyl pyrocarbonate (Sigma-Aldrich Corp. Ltd., MO, USA.) was used to dissolve siRNA to reach a 20 μmol·L^−1^ concentration. The transfection mixture per well was prepared using 5 μL Endofectin (GeneCopoeia Inc., USA) and 5 μL *GLUT1* siRNA or noncoding siRNA. After transfection for 48 h, the *GLUT1*^−/−^ BMDM was cultured as described above.

### Statistical analysis

The figures are representative of all data (*n* = 10). Data are presented as mean ± SD. Statistical analysis was done using Graphpad Prism software (Version 8.0.0). Significance was determined using Student’s *t-*test or ANOVA, as appropriate. *P*-values < 0.05 were considered statistically significant.

## Supplementary information

Supplementary table

Extended data figure 1

Extended data figure 2

Extended data figure 3

Extended data figure 4

Extended data figure 5

Supplemental figure legends
