# Supplementary material for: Diabetes fuels periodontal lesions via GLUT1-driven macrophage inflammaging
Source: Int J Oral Sci. 2021 Mar 24;13:11. doi: 10.1038/s41368-021-00116-6 (PMC7990943; doi:10.1038/s41368-021-00116-6)
Supplement: Supplementary file 1 — Supplementary table [file 41368_2021_116_MOESM1_ESM.docx]

**Supplementary Table 1**

**Relative mRNA Abundance of BMDMs in the different groups.**

| **Clust1** | **Clust2** |
| --- | --- |
| Tgfb2^†^  Adcy9^††^  Gnas^** ††^  Il12rb2^†^  Akt3^**^  Htr2b^††^  Phka2^**^  Igf1^††^  Eno2^*^  Plcb2^** ††^  Htr2a^††^  Ptger2^††^  Xylb^*^  Asic1^††^  Ptger4^††^  Pck2^* **^  Jun^†^  Adcy2^** ††^ | Map2k6^††^  Gapdh^*^  Il12b^†^  Pla2g4a^††^  Il1b^† ††^  H2-eb1^†^  Plcb4^** ††^  Sik1^**^  Nod2^†^  Il12rb1^†^  Cyp4a32^††^  Aldoc^*^  Slc2a1^**^ (Glut1)  Mapk8^††^  Prkce^††^  Plcb1^** ††^  Il1r1^††^  Il21r^†^  Adh7^*^  Il1rap^††^  Ugt1a2^*^  Il10^†^  Pgk1^*^  Il12a^†^  Il23r^†^  Il6^†^  Il1a^†^  Stat1^†^  Stat3^†^  Gys1^**^  Pfkp^*^  Il2rg^†^  Prkcb^††^  Pgm2^*^  Acss2^*^  Tpi1^*^  Ugt1a6b^*^  Pfkl^* **^  Il4ra^†^  Tlr2^†^  Gpi1^*^  Stat4^†^  Akr1b8^* **^  H2-ab1^†^  Pygl^**^ |

The genes from top to bottom listed in the table was corresponding to the sequence of genes in heat map (figure 4).

^*^ Contribution to glycolysis/gluconeogenesis.

^**^ Contribution to glucagon signaling pathway.

^†^ Contribution to inflammatory disease.

^††^ Contribution to inflammatory mediator regulation of transient receptor potential (TRP) channels.

**Supplementary Table 2**

**The typically early/late/metabolic SASPs*.**

| **early SASP** | Interleukin-1 β, IL-1β; |
| --- | --- |
| **late SASP** | c-c motif chemokine ligand 2, CCL2; |
|  | matrix metallopeptidase 3, MMP3. |
|  | IL-6; |
| **metabolic SASP** | transforming growth factor, TGF-β |
|  | IL-10; |

*Ref. De Cecco, M., Ito, T., Petrashen, A. P., Elias, A. E., Skvir, N. J., Criscione, S. W., . . . Sedivy, J. M. (2019). L1 drives IFN in senescent cells and promotes age-associated inflammation. *Nature, 566*(7742), 73-78. doi:10.1038/s41586-018-0784-9

**Supplementary Table 3**

**The SASP profiles shown in heatmap.**

| IL-1β | Interleukin-1 β |
| --- | --- |
| IL-6 | Interleukin-6 |
| IL-10 | Interleukin-10 |
| TNF-α | tumor necrosis factor-α |
| MMP-2 | matrix metalloproteinases-2 |
| MMP-8 | matrix metalloproteinases-8 |
| ICAM-1 | intercellular adhesion molecule-1, |
| M-CSF | macrophage colony stimulating factor |
| RAGE | receptor for advanced glycation end product |
| PP | pancreatic polypeptide |
| glucagon | / |
| leptin | / |
| Adi | adiponectin |
| OPG | osteoprotegerin |

**Supplementary Table 4**

**Materials.**

| **Antibody** | **Information** |
| --- | --- |
| GLUT1 | 1:2000 mouse mAb, ab40084, Abcam, Abcam Trading Co. Ltd., Shanghai, China |
| GAPDH | 1:1000 rabbit pAb, sc-25778, Santa Cruz Biotechnology, Inc, CA, USA |
| Rheb | 1:1000 mouse mAb, sc-271509, Santa Cruz Biotechnology, Inc, CA, USA |
| mTOR | 1:1000 rabbit mAb, #2983, Cell Signaling Technology, Massachusetts, USA |
| phospho‐mTOR (p‐mTOR) | 1:1000 mouse mAb, sc-293133, Santa Cruz Biotechnology, Inc, CA, USA |
| NF-κB | 1:1000 mouse mAb, sc-514451, Santa Cruz Biotechnology, Inc, CA, USA |
| phospho‐NF-κB (p-NF-κB) | 1:1000 mouse mAb, sc-136548, Santa Cruz Biotechnology, Inc, CA, USA |
| IL-1β | 1:1000 rabbit mAb, #31202, Cell Signaling Technology, Massachusetts, USA |
| p16 | 1:1000 mouse mAb, sc-166760, Santa Cruz Biotechnology, Inc, CA, USA |
| p21 | 1:1000 mouse mAb, sc-166630, Santa Cruz Biotechnology, Inc, CA, USA |
| **ELISA kit** |  |
| IL-1β | ZC37974, Zhuocai Bio., China |
| IL-6 | ZC-37988, Zhuocai Bio., China |
| MMP3 | ZC-38277, Zhuocai Bio., China |
| CCL2 | ZC-38588, Zhuocai Bio., China |
| TGF-β | ZC-39042, Zhuocai Bio., China |
| IL-10 | ZC-37962, Zhuocai Bio., China |
