# Supplementary figures and images for: Diabetes fuels periodontal lesions via GLUT1-driven macrophage inflammaging
Source: Int J Oral Sci. 2021 Mar 24;13:11. doi: 10.1038/s41368-021-00116-6 (PMC7990943; doi:10.1038/s41368-021-00116-6)

# Extended Data Fig. 1

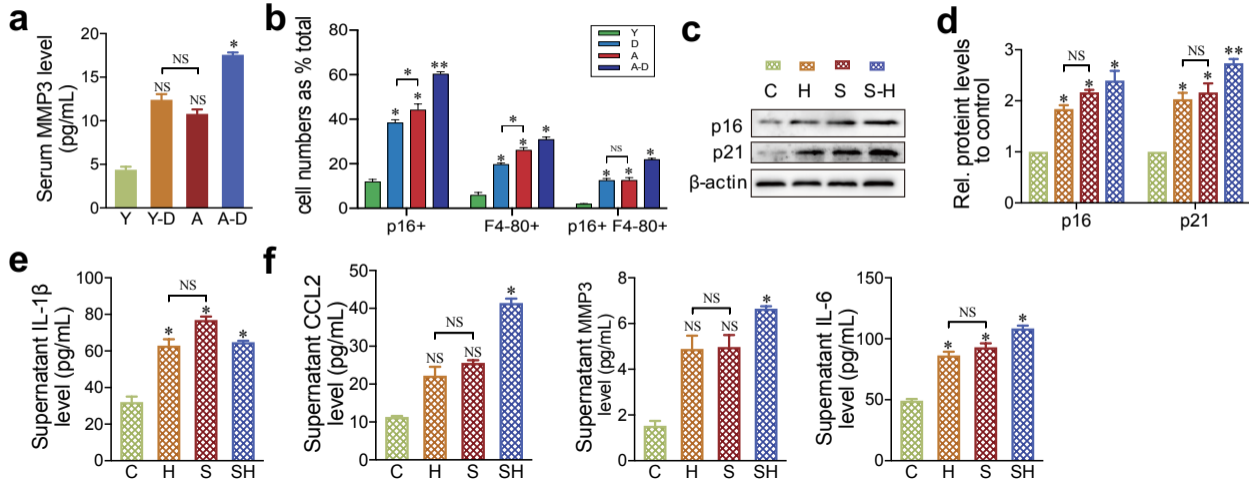

Supplement: Supplementary file 2 — Extended data figure 1 [file 41368_2021_116_MOESM2_ESM.pdf]

# Extended Data Fig. 2

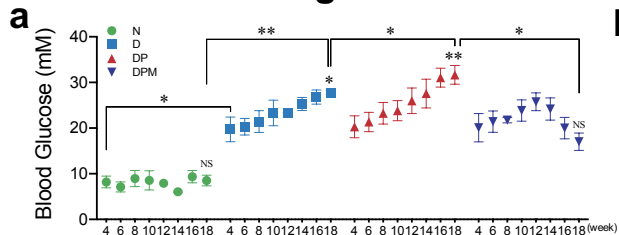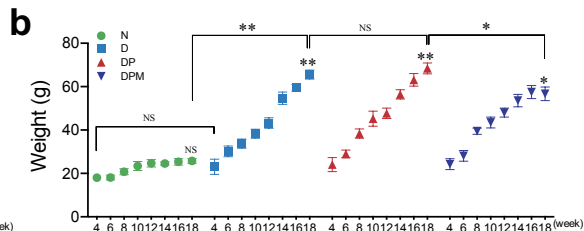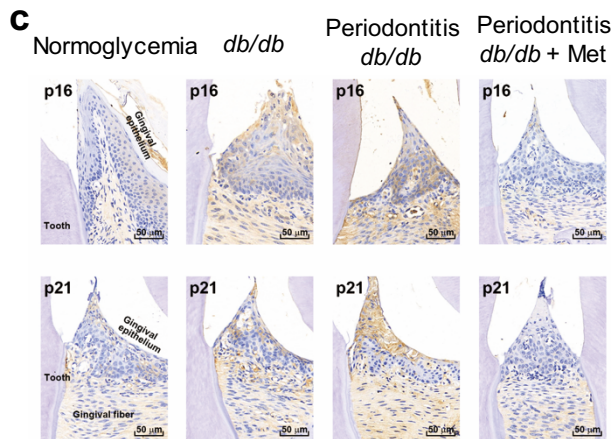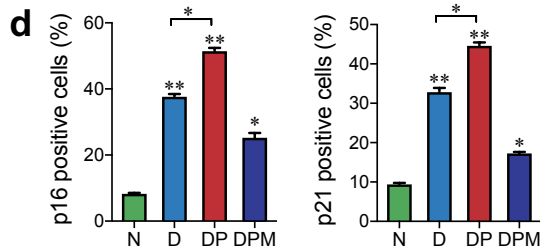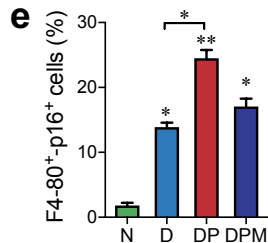

Supplement: Supplementary file 3 — Extended data figure 2 [file 41368_2021_116_MOESM3_ESM.pdf]

# Extended Data Fig. 3

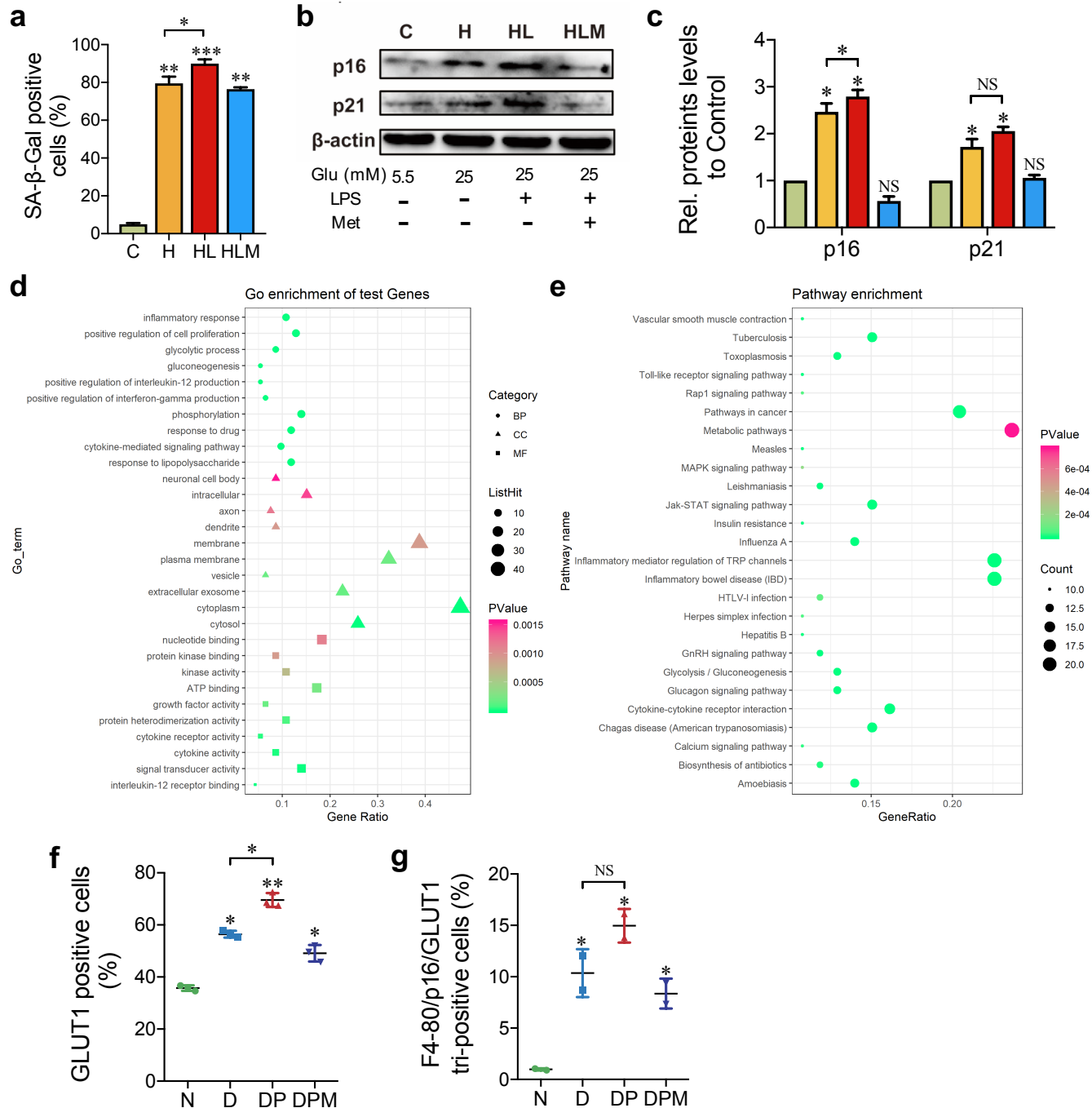

Supplement: Supplementary file 4 — Extended data figure 3 [file 41368_2021_116_MOESM4_ESM.pdf]

# Extended Data Fig. 4

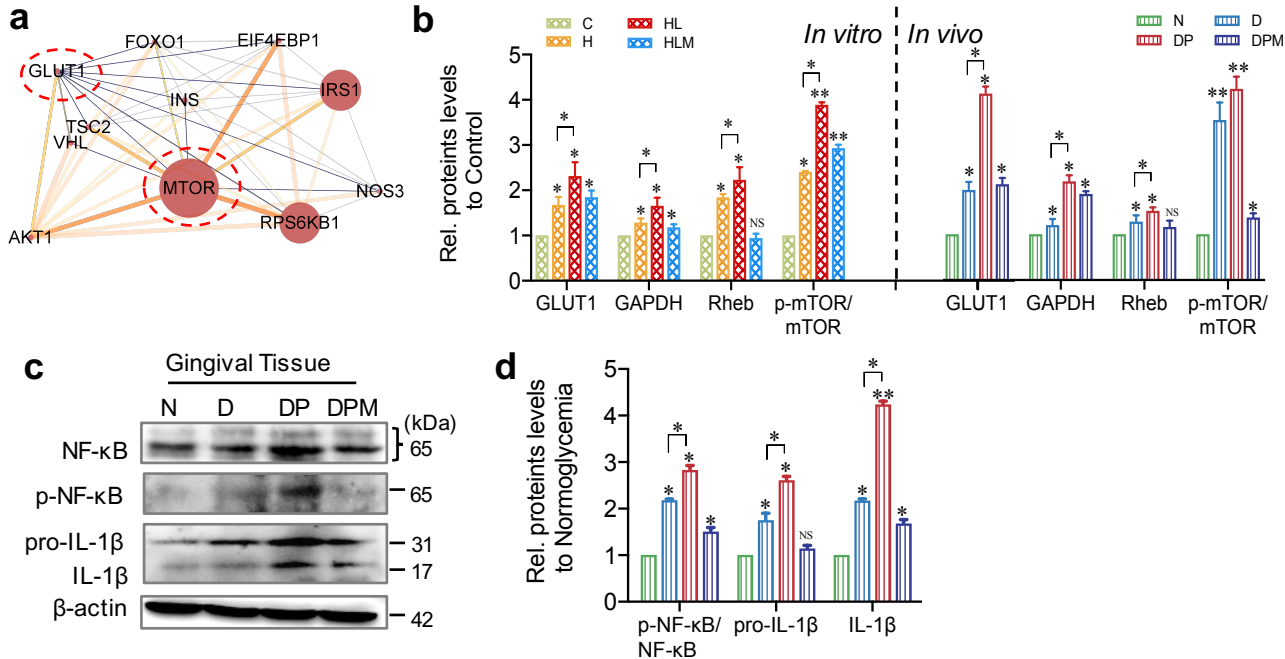

Supplement: Supplementary file 5 — Extended data figure 4 [file 41368_2021_116_MOESM5_ESM.pdf]

# Extended Data Fig. 5

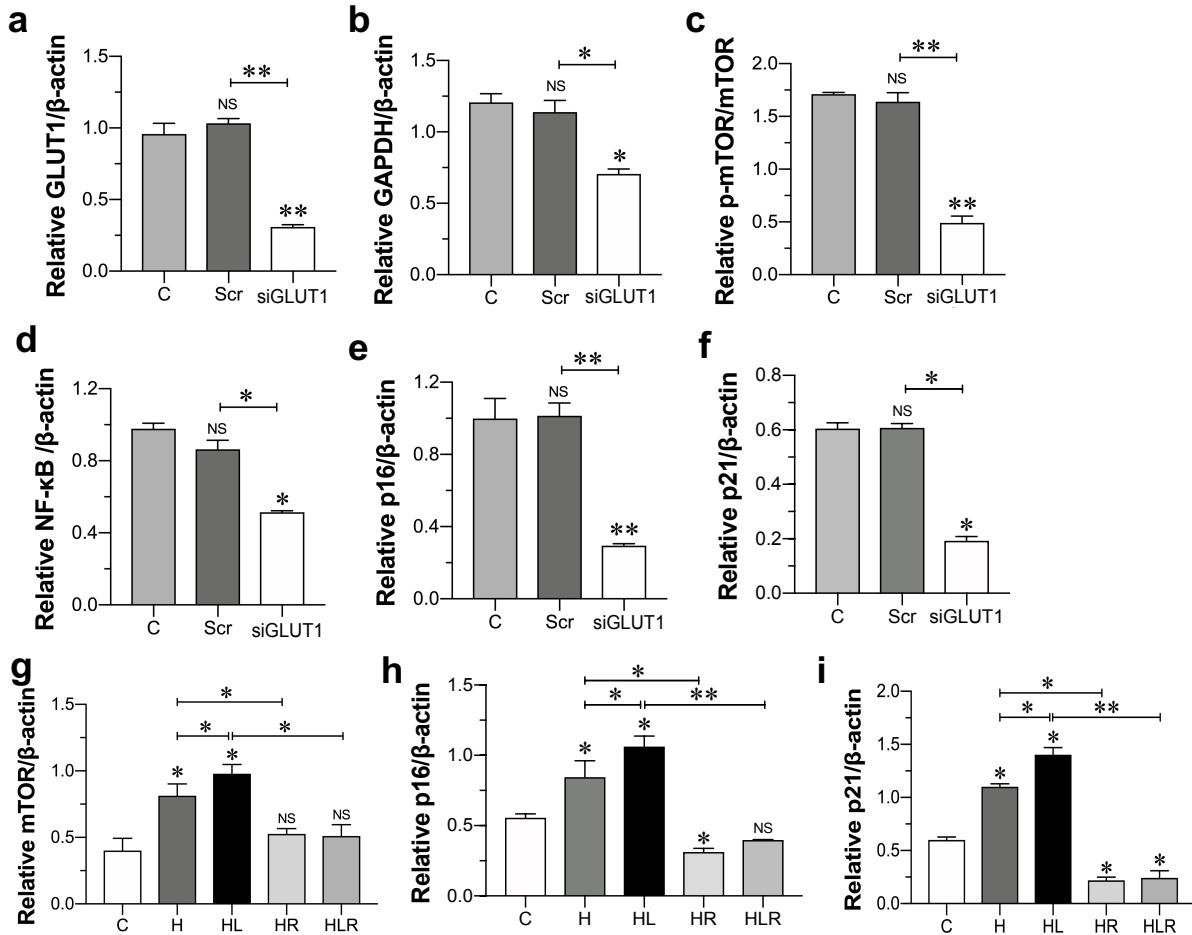

Supplement: Supplementary file 6 — Extended data figure 5 [file 41368_2021_116_MOESM6_ESM.pdf]
