# Supplementary material for: Diabetes fuels periodontal lesions via GLUT1-driven macrophage inflammaging
Source: Int J Oral Sci. 2021 Mar 24;13:11. doi: 10.1038/s41368-021-00116-6 (PMC7990943; doi:10.1038/s41368-021-00116-6)
Supplement: Supplementary file 7 — Supplemental figure legends [file 41368_2021_116_MOESM7_ESM.docx]

**Extended data figure 1**

**(a) Serum late SASP response.** The levels of MMP3 in diabetic and aged mice were detected by ELISA.

**(b)** Quantification of the abundance of p16^+^-macrophage (F4/80^+^) in periodontal tissues.

**(c)** Cell cycle modify *in vitro* senescent model. p16 and p21 were detected by western blot.

**(d)** Protein p16/p21 levels relative to β‐actin protein levels were assessed by densitometric analysis and expressed as percentage of the levels of control.

**(e)&(f)** The SASP responses *in vitro* senescent model, including early SASP (IL-1β) and late SASP response (CCL2, MMP3 and IL-6).

**Extended data figure 2**

**(a)** **Blood glucose-courses in young *db/db* mice.** The fasting blood glucose of mice was measured at 4 (baseline), 6 (1 wk after *p.g.* infection), 8 (1 wk after metformin treatment), 12, 16 and18 weeks old, respectively.

**(b)** **Weight-courses in young *db/db* mice.**

**(c)&(d) Induction of p16/p21 in young *db/db* mice.** Immunohistochemistry staining for p16/p21 in periodontal tissue of different groups and quantification of positive cells in the periodontal lesion area. Scale bars: 50 μm, 40×.

**(e)** Quantification of the abundance of p16^+^-macrophage (F4/80^+^) in periodontal tissues.

**Extended data figure 3**

**(a)** Quantification of SA-β-Gal positive cells in BMDM.

**(b)** The protein p16/p21 expressions *in vitro* were detected by western blot.

**(c)** Protein levels relative to β‐actin protein levels were assessed by densitometric analysis and expressed as percentage of the levels of control.

**(d)&(e)** GO and KEGG analysis of the differentially expression mRNA in high-glucose BMDM determined the roles of these co-expressed genes with a bubble chart. The mRNA function was predicted by GO functional annotation of co-expressed genes. Gene functions were classified into three subgroups namely biological process (BP), cellular component (CC) and molecular function (MF). GO terms with *p* <0.05 were selected and integrated using Venn analysis. P value and gene ratio were presented. KEGG analysis was performed to determine the involvement of co-expressed genes in different biological pathways.

**(f)** Quantification of GLUT1 positive cells detected by IHC staining in periodontal lesions.

**(g)** Quantification of triple-positive cells stained with GLUT1, F4/80 and p16 in the periodontal lesions.

**Extended data figure 4**

**(a)** The Protein-Protein Interaction Network (PPI) displayed the interaction between *GLUT1* and *mtor*.

**(b)** Protein GLUT1, GAPDH, Rheb, p-mTOR/mTOR levels of BMDM and the gingival tissues relative to β‐actin protein levels were assessed by densitometric analysis and expressed as percentage of the levels of control or wild‐type mice.

**(c)&(d)** The expressions of mTOR downstream protein in gingival tissues of different groups.

**Extended data figure 5**

**(a-f)** Quantification of proteins expression in *GLUT1 ^-/-^* BMDM relative to β-actin levels. **Control**, BMDM cultured in high-glucose medium (25 mmol/L glucose). **Scramble,** the non-targeted siRNA as a negative control.

**(g-i)** Quantification of proteins expression in BMDM with mTOR inhibition (rapamycin). **C**, BMDM cultured in low-glucose medium (5.5 mmol/L glucose); **H/HL**, BMDM cultured in high-glucose medium (25 mmol/L glucose) without or with LPS; **H-R/HL-R**, BMDM cultured in 12-h H/HL condition and then treated with 50 μg/L rapamycin.

* *p* < 0.05. ** *p* < 0.01. *** *p* < 0.001. NS, no significance. Repeated 3 times. Data are presented as the mean ± SD (n = 5 per group).
